# Loss of Guanylyl Cyclase C (GCC) Signaling Leads to Dysfunctional Intestinal Barrier

**DOI:** 10.1371/journal.pone.0016139

**Published:** 2011-01-31

**Authors:** Xiaonan Han, Elizabeth Mann, Shila Gilbert, Yanfang Guan, Kris A. Steinbrecher, Marshall H. Montrose, Mitchell B. Cohen

**Affiliations:** 1 Division of Gastroenterology, Hepatology, and Nutrition, Cincinnati Children's Hospital Medical Center, Cincinnati, Ohio, United States of America; 2 Department of Molecular and Cellular Physiology, University of Cincinnati, Cincinnati, Ohio, United States of America; Emory Unviersity, United States of America

## Abstract

**Background:**

Guanylyl Cyclase C (GCC) signaling via uroguanylin (UGN) and guanylin activation is a critical mediator of intestinal fluid homeostasis, intestinal cell proliferation/apoptosis, and tumorigenesis. As a mechanism for some of these effects, we **hypothesized** that GCC signaling mediates regulation of intestinal barrier function.

**Methodology/Principal Findings:**

Paracellular permeability of intestinal segments was assessed in wild type (WT) and GCC deficient (GCC−/−) mice with and without lipopolysaccharide (LPS) challenge, as well as in UGN deficient (UGN−/−) mice. IFNγ and myosin light chain kinase (MLCK) levels were determined by real time PCR. Expression of tight junction proteins (TJPs), phosphorylation of myosin II regulatory light chain (MLC), and STAT1 activation were examined in intestinal epithelial cells (IECs) and intestinal mucosa. The permeability of Caco-2 and HT-29 IEC monolayers, grown on Transwell filters was determined in the absence and presence of GCC RNA interference (RNAi). We found that intestinal permeability was increased in GCC−/− and UGN−/− mice compared to WT, accompanied by increased IFNγ levels, MLCK and STAT1 activation in IECs. LPS challenge promotes greater IFNγ and STAT1 activation in IECs of GCC−/− mice compared to WT mice. Claudin-2 and JAM-A expression were reduced in GCC deficient intestine; the level of phosphorylated MLC in IECs was significantly increased in GCC−/− and UGN−/− mice compared to WT. GCC knockdown induced MLC phosphorylation, increased permeability in IEC monolayers under basal conditions, and enhanced TNFα and IFNγ-induced monolayer hyperpermeability.

**Conclusions/Significance:**

GCC signaling plays a protective role in the integrity of the intestinal mucosal barrier by regulating MLCK activation and TJ disassembly. GCC signaling activation may therefore represent a novel mechanism in maintaining the small bowel barrier in response to injury.

## Introduction

Guanylyl cyclase C (GCC) is a transmembrane receptor for the endogenous peptides guanylin (GN) and uroguanylin (UGN) and for bacterial heat stable enterotoxin (ST) [1], [2]. GCC signaling plays a pivotal role in the regulation of intestinal fluid and electrolyte homeostasis [3]. Activation of GCC leads to increased intracellular cyclic GMP (cGMP) accumulation and activation of the cystic fibrosis transmembrane conductance regulator. Activation in response to the superagonist ST results in secretory diarrhea [4], [5]. In addition, GCC signaling regulates the renewal of the intestinal epithelium by restricting the proliferating cell cycle and promoting the transition from proliferation to differentiation along the crypt to villus axis [6], [7]. Deregulated GCC action is postulated to result in colorectal tumorigenesis and GCC expression is used as marker for human colorectal cancer metastases [8], [9]. Cross talk between activation of GCC signaling and c-src in colonic epithelial cells might also represent a feed-forward mechanism of cancer cell proliferation and disease progression in colorectal cancer [10]. Our group has reported that activation of GCC signaling pathway protects intestinal epithelial cells from acute radiation-induced apoptosis [11]. However, it remains unknown whether GCC directly mediates the regulation of intestinal epithelial barrier function.

Barrier function is highly regulated by tight junction proteins (TJPs), allowing the epithelium to control transmucosal permeability to solutes, water, and electrolytes [12], [13]. Amongst many components of TJs, occludin, junction adhesion molecule A (JAM-A), and claudins are membrane proteins that connect adjacent cells and build the intestinal barrier [13], [14]. Activation of actomyosin contraction, as assessed by phosphorylation of the myosin II regulatory light chain (MLC), regulates the assembly of TJPs [15]. Increased MLC kinase (MLCK) activity has been demonstrated to mediate intestinal epithelial barrier dysfunction induced by tumor necrosis factor α (TNFα) and interferon γ (IFNγ) [16], [17], [18]. Cytokine-induced STAT1 activation in IECs mediates the onset of intestinal diseases [19], [20]. Conversely, depletion or pharmacological inhibition of epithelial MLCK protected mice from TNFα-dependent intestinal epithelial barrier loss, and improved diarrheal symptoms [21].

Defects in intestinal barrier function have been implicated in the pathogenesis of a number of intestinal diseases, such as sepsis, inflammatory bowel disease (IBD) and irritable bowel syndrome (IBS). In our current studies, employing GCC and GCC ligand deficient mice, we demonstrate that GCC signaling is required for the maintenance of homeostatic intestinal barrier function, identifying a novel pathway as well as a new potential therapeutic target for intestinal barrier dysfunction.

## Results

### Loss of GCC signaling increases paracelluar permeability in small intestine

**Figure 1:**
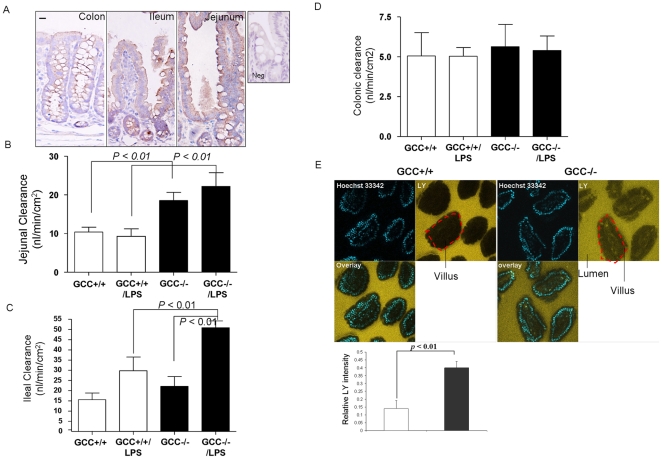
Loss of GCC signaling increases paracellular permeability in small intestine. A) GCC expression was detected in colon, ileum, and jejunum by immunohistochemistry (IH), inset is a negative control (Neg); original magnification, ×400, bar = 50 µm, n = 5. B, C & D) Jejunal, ileal and colonic paracellular permeability was determined using an everted gut sac in WT (GCC+/+) and GCC knock out (GCC−/−) mice with and without LPS (1 mg/kg) challenge, n = 10. E) Permeability to Lucifer Yellow (LY) was measured in jejunum from GCC+/+ and GCC−/−, n = 5. Relative intensity was determined by the ratio of LY intensity inside villi versus luminal side. Results are expressed as the mean ± SEM.

As previously shown [8], [22], [23], we confirmed GCC expression throughout all intestinal segments. However, by qualitative immunohistochemistry, GCC was mainly expressed in IECs (Fig 1 A). We next determined paracellular permeability of intestinal segments in GCC−/− and WT mice. We found that jejunal paracellular permeability to FD4 was significantly higher in GCC−/− mice under basal conditions; this was not seen in ileum and colon (Fig 1 B, C and D). Analyzing LY uptake into jejunal villi of live mice by confocal microscopy, we consistently found that the integrity of jejunal epithelia was disrupted in GCC−/− mice, as shown by significantly increased LY inside villi. This was not apparent in the ileum and colon (Fig 1 E and data not shown). Together, these results demonstrate that loss of GCC signaling in the jejunum leads to mucosal barrier dysfunction. In addition, under basal conditions, compensation for loss of GCC precludes barrier defects in the ileum and colon.

### GCC−/− mice are predisposed to LPS-induced intestinal injury

**Figure 2:**
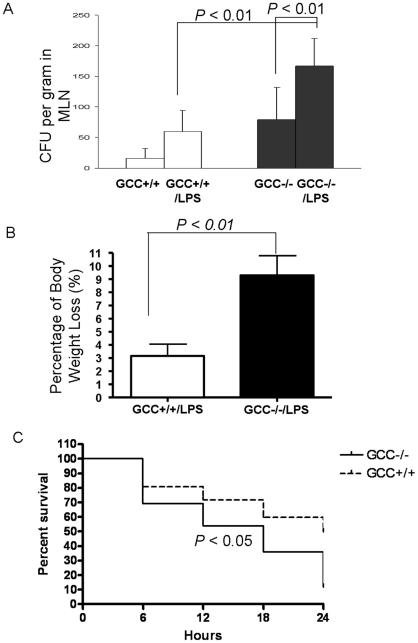
GCC−/− mice are predisposed to lipopolysaccharide (LPS) induced intestinal injury. GCC+/+ and GCC−/− mice were challenged with LPS (1 mg/kg) for 12 hrs, and bacterial translocation in MLN (A) and percentage of body weight loss (%) (B) were determined, n = 10. C) Mortality of GCC−/− mice was determined after 24 hr-LPS (4 mg/kg) challenge, n = 16. Results are expressed as the mean ± SEM.

To further determine the function of GCC signaling in intestinal barrier, we challenged wild type (GCC+/+) mice and GCC−/− mice with a non-lethal dose of LPS (1 mg/kg), which has been reported to cause a mild and reversible alteration in intestinal barrier function by promoting bacterial translocation and cytokine secretion [24]. LPS challenge (12 hr) did not increase permeability in the jejunum of GCC+/+ mice or the already elevated permeability in GCC−/− mice (Fig 1B); however, we found there was remarkably elevated permeability in the ileum of both genotypes after 12-hr LPS challenge and the increase was significantly higher in GCC−/− mice compared to baseline (Fig 1B & C). Consistently, a significantly higher amount of bacteria translocated to mesenteric lymph nodes (MLN) in GCC−/− mice relative to GCC+/+ mice after 12-hr LPS challenge (Fig 2A), demonstrating that loss of GCC leads to ileal barrier dysfunction after LPS challenge; also, GCC−/− mice consequently lost a significantly higher percentage of body weight than WT mice (*p*<0.01, Fig 2 B). Furthermore, upon increasing the LPS dose to 4 mg/kg, we found that 90% of the GCC−/− mice did not survive by 24 hrs after LPS challenge, (*p*<0.05, see Fig 2 C), suggesting that loss of GCC leads to catastrophic intestinal barrier failure and results in death due to LPS-induced sepsis.

### Loss of GCC signaling increases MLC phosphorylation in IECs and disrupts TJP assembly in small intestine

**Figure 3:**
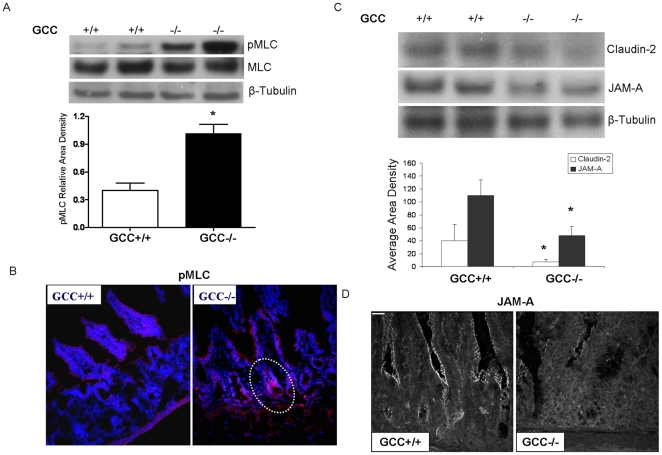
Loss of GCC signaling increases MLC phosphorylation in IECs and disrupts TJP assembly in small intestine. Jejunal IECs were isolated with EDTA, and total protein was extracted from jejunal IECs, A) MLC abundance and phosphorylated MLC (pMLC) were measured by Western blot, densitometry results normalized to total MLC, n = 5. B) pMLC distribution was determined with immunofluorescence; dotted lines encircle staining of pMLC, n = 5. C) Total protein was extracted from jejunal tissue, and JAM-A and Claudin-2 abundance was determined by Western blot, n = 5. D) JAM-A distribution was determined with immunofluorescence, n = 5, original magnification, ×400, bar = 50 µm. Signal intensity was determined by densitometry. Quantitated western blot results are shown as the mean ± SEM. * *p*<0.05 versus GCC+/+ mice.

TJPs regulate intestinal paracellular permeability, controlling the penetration of pathogens and allergens to the submucosa [12], [13]. We first studied an up-stream kinase of TJP assembly, MLCK which can be viewed as a final common pathway of acute tight junction regulation in response to a broad range of immune and infectious stimuli [16], [25]. We found that loss of GCC led to a significantly increased cellular abundance of pMLC in IECs detected by both immunoblot and immunofluorescence (Fig 3A & B). Subsequently, immunoblotting demonstrated that JAM -A and Claudin-2 were significantly reduced in jejunum from GCC−/− mice (Fig 3C). We also found a consistently reduced JAM-A abundance in GCC−/− crypts using confocal immunofluorescence microscopy (Fig 3D). This indicates that GCC signaling directly regulates the production and/or assembly of TJPs like JAM-A and claudin-2. Together, these data strongly suggest that GCC signaling mediates regulation of intestinal barrier function by regulation of TJP assembly.

### Loss of UGN leads to increased paracellular permeability in small intestine

**Figure 4:**
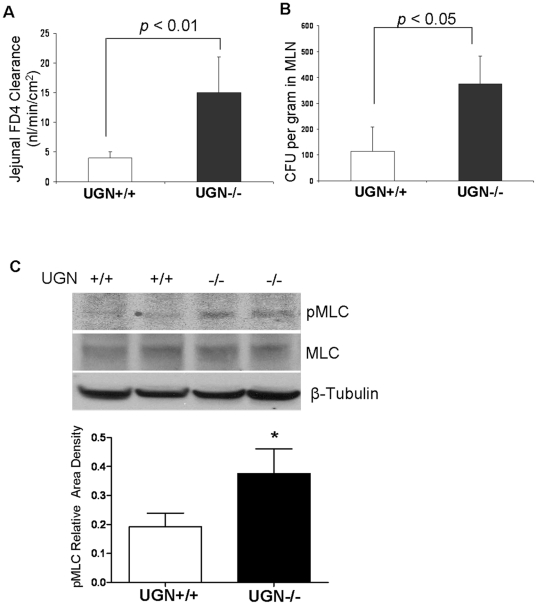
Loss of uroguanylin leads to increased paracelluar permeability in small intestine. Jejunal paracellular permeability was determined by assessing flux of FD4 with an everted gut sac (A) and bacterial translocation in mesenteric lymph nodes (MLNs) (B) in WT (UGN+/+) and uroguanylin deficient mice (UGN−/−), n = 10. Jejunal IECs were isolated with EDTA, C) Myosin light chain (MLC) abundance and phosphorylated MLC (pMLC) were measured with WB, densitometry results normalized to total MLC, n = 4. Results are expressed as the mean ± SEM. * *p*<0.05 versus GCC+/+ mice.

To test whether the role of GCC in maintaining barrier function was ligand-dependent, we studied permeability in the jejunum of UGN−/− mice. UGN−/− mice are also relatively deficient in GN [3]. We found that UGN−/− mice demonstrated significant jejunal barrier dysfunction characterized by increased paracellular permeability and bacterial translocation to MLN along with increased MLC phosphorylation under basal conditions (see Fig 4A, B & C), suggesting that intestinal UGN and GN are also required for intestinal barrier homeostasis.

### LPS exaggerates cytokines production in GCC−/− intestinal mucosa

**Figure 5:**
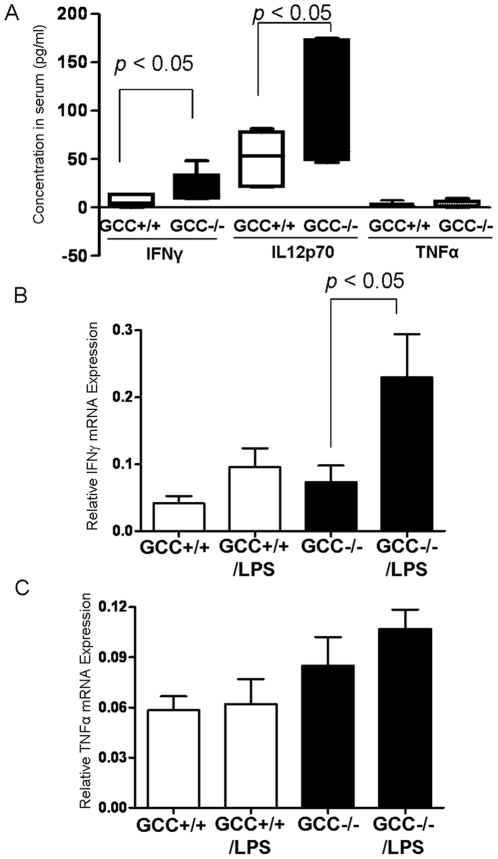
LPS exaggerates cytokine production in GCC−/− intestinal mucosa. A) Levels of IFNγ, IL12p70, and TNFα were determined in the circulation, using Bioplex™, n = 8. B & C) TNFα and IFN-γ mRNA levels were determined by real-time PCR in jejunal tissue either under basal condition or following LPS challenge, n = 6. Results are shown as the mean ± SEM.

To further pursue the mechanism of increased barrier dysfunction in GCC−/− mice, we measured levels of cytokines in the circulation. We found that IFN-γ as well as IL12p70, but not TNFα, were significantly elevated in the peripheral circulation in GCC−/− mice at baseline (Fig 5A). Using quantitative PCR, we found no significant difference between genotypes in IFNγ or TNFα levels in the small intestine at baseline. However, a non-lethal dose of LPS significantly upregulated IFNγ mRNA expression in GCC−/− mice, while TNFα levels were not significantly different (Fig 5B & C). This suggests that GCC null mice have a deregulated immune function, resulting in susceptibility to LPS induced intestinal injury.

### Loss of GCC signaling activates IFN-γ:MLCK pathway in IECs

**Figure 6:**
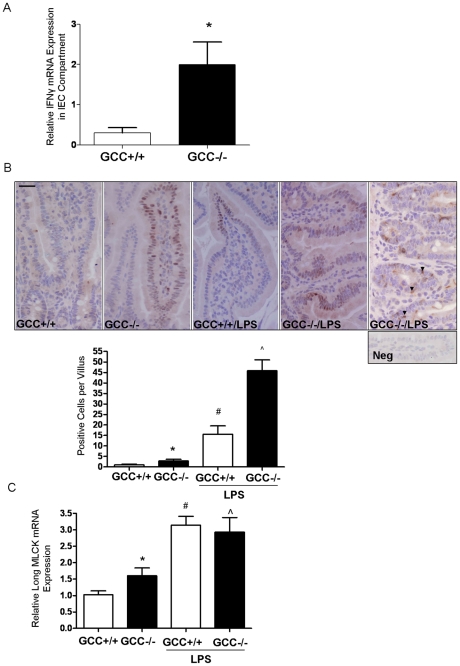
Loss of GCC signaling activates IFN-γ:MLCK pathway in IECs. A) Jejunal IECs were captured by LCM, RNA isolated and the level of IFNγ mRNA was assessed by real-time PCR. n = 7. B) Paraffin-embedded jejunal tissue was immunostained by pSTAT1, n = 5, original magnification, ×400, bar = 50 µm. pSTAT1 positive cells were counted by a semi-quantitative method and expressed as average positive cells per villus. Arrows indicate pSTAT1 staining of intraepithelial lymphocytes in tissue from GCC−/− mice. C) Long MLCK mRNA levels were determined by real-time PCR in jejunal tissue either under basal condition or following LPS challenge, n = 6. Results are shown as the mean ± SEM. * *p*<0.05 versus WT group; # *p*<0.05 versus LPS treated WT group and ∧ versus LPS treated GCC−/− group.

Employing laser capture microdissection (LCM), we isolated jejunal IECs. We found there was a dramatically elevated IFN-γ mRNA level in the IEC compartment in GCC−/− mice at baseline compared to wild type (Fig 6A). STAT1 activation is an important mediator of IFN-γ signaling, reflecting intestinal mucosal immune response and inflammation [19], [26]. Concomitant with increased levels of IFN-γ in IEC compartment, immunohistochemistry results showed that there was increased phosphorylated STAT1 (pSTAT1) staining in jejunal IECs which was sparsely distributed in a patchy fashion (Fig 6B). After 12 hr-LPS challenge, STAT1 activation in IECs was greatly increased and exhibited a more pervasive distribution in GCC−/− mice (Fig 6B). This was confirmed to be a significant increase by a semi-quantitative counting of pSTAT1 in villus IECs (Fig 6B). Interestingly, flow cytometry analysis indicated that CD3^+^ intra-epithelial lymphocytes (IEL) were significantly increased in the intestinal epithelial compartment of GCC−/− mice (1.85±0.15% in WT versus 2.86±0.4% in GCC−/− mice, *p*<0.01, n = 7); we also observed there was a fair amount of pSTAT1 positive IELs in LPS-treated GCC−/− (Arrows, Fig 6B). These data suggest that jejunal epithelial barrier has been severely damaged in GCC−/− mice, and the infiltrated CD3^+^ IELs might be the resource of elevated IFNγ in IEC compartment. As IFN-γ primes intestinal epithelia to respond to TNF-α and LIGHT which are associated with induction of the epithelial (long) isoform of MLCK [18], [27], we employed real time PCR to quantitate levels of expression in wild type and GCC−/− mice. We found long MLCK mRNA levels were significantly up regulated in GCC deficient jejunum at baseline, and were potentiated by LPS challenge both in WT and in GCC−/− mice. Together, GCC may mediate the regulation of IEC barrier specifically through an IFN-γ:MLCK pathway.

### Reduction of GCC signaling leads to hyperpermeability in IEC monolayer

**Figure 7:**
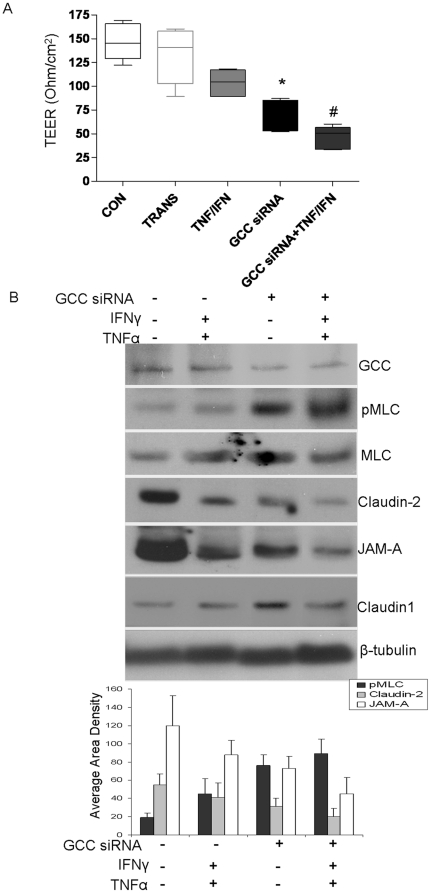
Reduction of GCC signaling decreases membrane-associated TJPs in IEC monolayer. A) Paracellular permeability in post-confluent Caco-2 cell monolayers was assessed by TEER in the presence or absence of GCC siRNA, and IFNγ (10 ng/ml) and TNFα (10 ng/ml), * *p*<0.01 versus TRAN (transfection reagents only) and ^#^ *p*<0.01 versus IFNγ and TNFα treated control cells, n = 5. Results are shown as the mean ± SEM. B) Total protein was extracted from post-confluent Caco-2 cell monolayers with and without IFNγ (10 ng/ml) and TNFα (10 ng/ml) treatment. JAM-A, Claudin-1 & 2, MLC, and pMLC were assessed by Western blot, signal intensity was determined by densitometry. Results representative of five independent experiments are shown and quantitated western blot results are expressed as the mean ± SEM.

We grew HT-29 IEC monolayers on Transwell filters and used RNA interference to achieve approximately 70% knockdown of GCC expression. We observed that paracellular permeability in post-confluent HT-29 cell monolayers, assessed by the apical-to-basolateral flux of FD4, was markedly increased by knocking down GCC expression (Figure S1, *p* = 0.02). Consistently, TEER was also reduced in GCC knock-down monolayers, compared to transfection reagent control without siRNA (*p* = 0.006, see Figure S2). Similar results were obtained using the Caco-2 intestinal cell line as well (Fig 7 and data not shown). These data indicate that IEC monolayer permeability could be increased by inhibiting GCC expression *in vitro*, analogous to results obtained with the GC-C knockout mouse.

### Reduction of GCC signaling up regulates MLC phosphorylation and decreases TJPs in IEC monolayer

To mimic *in vivo* studies, we combined IFNγ (10 ng/ml) with TNFα (10 ng/ml) to stimulate the Caco-2 monolayers for 48 hr [17]. Monolayer hyperpermeability, induced by cytokines, was significantly enhanced by GCC knock-down compared to cytokine-treated controls (*p*<0.01, Fig 7A). We next examined MLC phosphorylation and the abundance of TJPs in Caco-2 monolayers after GCC knock down. TNFα and IFNγ cause TJ disruption and epithelial barrier loss by activating MLCK [17], [18]. We first measured pMLC and found that pMLC was upregulated by GCC knock down, which was further enhanced by IFNγ and TNFα treatment compared to controls (Fig 7B). We then found that JAM-A and Claudin-2, membrane-associated TJPs, were significantly reduced in IEC monolayers with GCC knock down; this was potentiated by IFNγ and TNFα administration (Fig 7B). Interestingly, we did not find apparent alterations of other components of TJPs, for example, claudin-1. Taken together, these data further indicate that GCC signaling may mediate regulation of intestinal epithelial barrier function directly through affecting TJP assembly.

## Discussion

Original descriptions of GCC null mice were marked by a paradoxical lack of an obvious phenotype and we and others suggested that the function of GCC would only be revealed by systemic study and perturbation of gastrointestinal function [4], [28]. This led to the establishment of roles for GCC in regulation of IEC proliferation, apoptosis, and migration [6], [9], [11]. Epithelial barrier function is a crucial component of gut homeostasis, and dysregulation contributes to the pathogenesis of many intestinal diseases. In this paper, we have shown that jejunal permeability was increased in GCC−/− and UGN−/− mice compared to WT. GCC−/− mice exhibited ileal hyperpermeability and greater bacterial translocation after LPS challenge, accompanied by increased IFNγ levels. The level of phosphorylated MLC in IEC was significantly increased in GCC−/− and UGN−/− mice compared to WT; Claudin-2 and JAM-A expression in TJs were reduced in GCC deficient IEC. GCC knockdown in IEC monolayers was associated with increased permeability under basal conditions and enhanced IFNγ induced hyperpermeability in IEC monolayers. Our data strongly suggest that GCC signaling plays a role in the integrity of the intestinal mucosal barrier by regulating epithelial MLC phosphorylation and TJ assembly.

Functional TJP strands are located between polarized epithelial cells and characterize highly-differentiated gastrointestinal epithelial cells [29]. GCC is highly expressed in differentiated enterocytes. Waldman and co-workers have shown that loss of GCC is associated with changes in IEC homeostasis, including increased proliferation in the crypt, increased migration along the crypt-villus axis, and increased apoptosis [6], [9], [30]. The magnitude of these differences decreased from duodenum to colon and parallels the level of hyperpermeability that we saw in different segments of the intestinal tract, with highest disruption of barrier function in the jejunum. It is possible that the changes in barrier function and TJPs that we observed may contribute at least partially to the alteration of homeostatic processes in GCC−/− intestine. Dedifferentiated IECs can lead to immature production of TJPs, and loss of contact inhibition [31]. Our results show that loss of GCC signaling reduced JAM-A and Claudin-2 *in vivo* and *vitro*, which have been determined to be associated with tumor progression [32], [33]. Most recently, GCC activation was found a polar pattern of the effects on ion transport; GCC mucosal activation resulted in a potent cGMP-chloride secretion, which may add to its role in the intestinal barrier [34].

GN and UGN are expressed in the highly-differentiated compartment along the crypt-villus axis, associated with the transition from proliferation to differentiation [35]. They exhibit a gradient of expression along the length of the gastrointestinal tract with UGN levels highest in the proximal intestine and GN levels highest in the colon [3]. Consistently, we found that UGN−/− mice, which also exhibit a significant decrease in intestinal GN protein [3], had a dysfunctional jejunal barrier function at baseline. Together, this suggests that intestinal UGN and GN as well as their signaling via GCC are required for the maintenance of small bowel barrier function. Levels of cGMP are reduced by 50–75% in the intestines of both GC-C−/− and UGN−/− mice [3], [11] and this reduction may provide a basis for loss of TJ function that remains to be investigated.

IFN-γ selectively increases epithelial permeability to large molecules by direct alteration of TJP assembly [36], [37]. STAT-1 is an important signaling molecule for IFN-γ; and STAT1 activation in IECs leads to downstream proinflammatory gene expression, predisposing IECs to injury [38], [39]. In our studies, we found that GCC signaling is involved in a complicated modulation of gut mucosal immunity. An increased level of cytokines (IFNγ and IL12p70) was detected in GCC knock out mice at baseline accompanied by significantly elevated jejunal permeability, MLCK expression and STAT1 activation in IECs. An important mechanism through which IFNγ drives barrier dysfunction is by increasing expression of TNF and LIGHT receptors on epithelial cells and sensitizing the IEC monolayer to cytokine stimulation [17], [18]. Low dose LPS challenge resulted in a further disruption of barrier function in the ileum of GCC null mice along with significantly elevated luminal bacterial translocation. The barrier dysfunction predisposed GCC null mice to LPS induced sepsis and organ dysfunction, and subsequently resulted in increased mortality upon high dose LPS challenge. Our data also indicated that IFNγ mRNA expression and IELs was elevated in LCM-captured jejunal IEC compartment, suggesting that intestinal barrier dysfunction in GCC deficient mice is maintained by a continuous immune activation. These data indicates that loss of GCC signaling may lead to a dysregulation of the mucosal immune system, and triggers intestinal barrier dysfunction and immune activation.

Primary pathophysiologically relevant intestinal epithelial barrier dysfunction can broadly activate mucosal immune responses and accelerate the onset and severity of immune-mediated colitis, but is not sufficient for intestinal disease [25], [40]. Cytokine-induced epithelial barrier dysfunction can be mediated by increased MLCK expression and subsequent myosin II regulatory light chain (MLC) phosphorylation; TNFα, IFNγ, and LIGHT (a member of the TNFα superfamily) can cause MLCK-dependent barrier dysfunction [21], [41]. Furthermore, MLCK upregulation is correlated with IBD disease activity, also suggesting that it may contribute to barrier dysfunction in intestinal disease [16]. Our data confirmed that loss of GCC signaling led to increased MLC phosphorylation, MLCK mRNA expression and IEC barrier dysfunction in mice. In comparison, our GCC knockdown studies in IEC monolayers highlight an increase in permeability accompanied by increased phosphorylation of MLC due only to decreased levels of GCC. However, the manner in which GCC signaling mediates the regulation of MLCK activity needs to be explored in the future. Together, loss of GCC signaling leads to the activation of IFN-γ:MLCK pathway in IECs and may be an important initiating event that leads to barrier dysfunction, followed by pro-inflammatory factor production and a predisposition to LPS-induced injury.

We found that reduced JAM-A and Claudin-2 abundance was consistently associated with loss of GCC in both GCC deficient mice and in GCC knock down IEC monolayers. JAM-A has been demonstrated to regulate junctional assembly through recruiting and binding these proteins to its intracellular C-terminus in order to colocalize junctional proteins with the nascent junctions [42]. JAM-A null mice exhibit increased intestinal mucosal permeability, and JAM-A has been determined to regulate epithelial permeability, inflammation, and proliferation [43]. Aberrant expression of Claudin-2 has been linked to SAMP1/YitFc (SAMP) mice, that develop chronic ileitis [44]. Claudin-2 can convert “tight” tight junctions into leaky ones, and it was identified as a cation-selective paracellular channel [45]. Upregulation of pore-forming claudin 2 leads to altered tight junction structure and pronounced barrier dysfunction in mild to moderately active Crohn's disease [46]. Conversely, reduced levels of claudin-2 or JAM-A may also lead to disrupted barrier function [36]. Therefore, GCC signaling may be relevant to regulation of intestinal barrier function directly through interacting with TJPs. The precise mechanisms elucidating how GCC signaling is involved in the regulation of TJPs is the basis for ongoing investigation.

Disruption of intestinal barrier function leading to mucosal inflammation and immune activation may be a key factor in the pathogenesis of several diseases, including sepsis, IBD and IBS [47], [48], [49]. IBS is characterized by an increased small bowel paracellular permeability and an increased load of luminal bacteria [48]. Linaclotide (MD-1100), a GCC agonist, has been shown in animal studies to stimulate intestinal fluid secretion and transit, but not in GCC null mice [50], [51]. Linaclotide improved bowel habits and symptoms of IBS patients with chronic constipation although the mechanisms of action downstream of cGMP are uncertain [51]. Our studies for the first time identify a novel GCC∶MLCK∶TJP pathway that regulates intestinal barrier function. Therefore, augmenting intestinal GCC activation may represent a novel approach for restoring mucosal barrier function in intestinal disorders.

## Materials and Methods

### Materials

All chemicals and antibodies were purchased from Sigma-Aldrich (St. Louis, MO) unless otherwise noted. Antibodies specific for MLC and phosphorylated MLC (serine 19) (pMLC) and STAT1 (pSTAT1) were from Cell Signaling Technology (Danvers, MA). Antibodies specific for JAM-A, Claudin 1, and 2 were from Zymed (Carlsbad, CA). Antibody for GCC was from FabGennix (Frisco, TX). ON-TARGET plus SMARTpool for human *GUCY2C* was purchased from DHARMACON (Chicago, IL).

### Animal resources and maintenance

Animal studies were approved by the Cincinnati Children's Research Foundation (CCRF) Institutional Animal Care and Use Committee (Protocol # 8E03019). GCC and UGN deficient mice (GCC−/− and UGN−/−) have previously been described [3], [28]. Mice were inbred for 10 generations to C57BL/6 and maintained in specific pathogen free conditions. To induce a systemic inflammatory response, mice were injected intraperitoneally with *Escherichia coli* (strain O111:B4, Sigma) lipopolysaccharide (LPS, 1 mg/kg or 4 mg/kg) in 0.5 mL of phosphate-buffered saline (PBS). Groups of mice were sacrificed 12 hrs after injection of LPS for intestinal permeability assay or 24 hrs after injection for survival study [24].

### Measurement of paracellular intestinal permeability and bacterial translocation

Jejunal, ileal and colonic paracellular permeability to the fluorescent tracer fluorescein isothiocyanate-dextran with a molecular mass of 4,000 Da (FD-4) was determined using an everted gut sac method [52]. Fluorescence was measured using a fluorescence spectrophotometer (Biotek Instruments, VT) at an excitation wavelength of 492 and an emission wavelength of 515 nm. Permeability was expressed as the mucosal-to-serosal clearance of FD-4. Bacterial translocation to mesenteric lymph nude (MLN) was determined as previously described [52].

### *In vivo* measure of local villus permeability

Methods are based on those described previously [53], [54]. Briefly, after anesthesia, ∼1 cm of jejunum was exteriorized, slit open, and the mucosal surface rinsed with saline. Cell nuclei were stained with Hoechst 33342 (2 mg/kg bw. Invitrogen, Eugene OR) and imaged with 2-photon microscopy (Zeiss LSM510 NLO, Jena Germany). Jejunal permeability was monitored by adding 50 µM Lucifer Yellow (LY; CH lithium salt, Molecular Probes) to the superfusate, and confocal fluorescence imaging (458 nm excitation, 505 nm emission). Post-acquisition image analysis used Metamorph 7 (Molecular Devices, Downingtown, PA) and ImageJ (NIH, Bethesda, MD). The intensity of luminal and tissue LY fluorescence was measured in appropriate regions and expressed as the tissue/lumen ratio.

### Immunoblotting (IB), Immunohistochemistry (IH) and immunofluorescence (IF)

Isolated jejunal IECs, jejunal tissue as well as cultured cells were saved. Total cellular protein extracts and cytosolic protein were prepared using cold RIPA buffer and the NE-PER kit per the manufacturers' recommendations (Pierce, Rockford, IL). Expression of claudin1 & 2, junction adhesion molecule (JAM-A) and MLC, and pMLC abundance were detected in total protein and in isolated jejunal IECs. Band intensities were quantified as mean area density using ImageQuant (Molecular Dynamics, Sunnyvale, CA). pMLC was expressed as relative area density corrected by MLC bands intensity. Frozen tissue sections from mouse jejunum (4 µm) were prefixed in paraformaldehyde. Tissue sections were labeled with pMLC and JAM-A. 4′,6-diamidino-2-phenylindole dihydrochloride (DAPI) was used for nuclear counterstaining following FITC-conjugated or TRITC-conjugated goat anti-rabbit secondary antibodies. GCC expression and phosphorylation of STAT1 were examined in paraffin embedded intestinal sections using VECTASTAIN Elite ABC system (Vector lab, Burlingame, CA). pSTAT1 positive cells were counted by a semi-quantitative method and expressed as average positive cells per villus. Images were captured using a Zeiss microscope and Axioviewer image analysis software (Deutschland, Carl Zeiss Corp, Germany) [52], [55].

### Laser Capture Microdissection (LCM)

Briefly, approximately 200 crypts and adjacent surface epithelial cells from jejunum were captured by a Veritas Microdissection System (Molecular Devices, CA); RNA was isolated with a PicoPure RNA Isolation kit (Arcturus) using our published methods [55], [56]. The quality and concentration of RNA was measured by NanoDrop (Thermo Fisher). Total RNA (200 ng) was used to reversely transcribe to cDNA followed by a SYBR Green real-time PCR on the Mx4000 multiplex quantitative PCR instrument (Stratagene).

### Real-time PCR

Total RNA was isolated from frozen tissue using Tri Reagent (Molecular Research Center, Inc., Cincinnati, OH) according to the manufacturer's protocol. RNA samples were treated with DNase I (Ambion, Austin, TX) and reverse-transcribed (2 µg) using random decamers (RETROscript, Ambion). PCR reactions using specific gene primers were performed with Brilliant II SYBR Green QPCR mix (Stratagene, La Jolla, CA) in the Mx3000p thermocycler (Stratagene). A relative amount for each gene examined was obtained from a standard curve generated by plotting the cycle threshold value against the concentration of a serially diluted RNA sample expressing the gene of interest. This amount was normalized to the level of β actin RNA. Primer sequences [57], [58] are listed in the Table 1.

**Table 1:** PCR Primers.

Primers	Sequences
β actin	Forward: 5′- CCCTAAGGCCAACCGTGAA -3′Reverse: 5′- CAGCCTGGATGGCTACGTACA -3′
TNFα	Forward: 5′ - AATGGCCTCCCTCTCATCAGTT - 3′Reverse: 5′ – CCACTTGGTGGTTTGCTACGA - 3′
IFNγ	Forward: 5′ - GGCTGTCCCTGAAAGAAAGC - 3′Reverse: 5′ - GAGCGAGTTATTTGTCATTCGG - 3′
Long MLCK	Forward: 5′ - AAGTCATGGATGGAAGCCAGGTCA- 3′Reverse: 5′ - AATCTCATTCCCGTCGTGAAGCCA- 3′

### RNA interference and IEC monolayer permeability assay [59], [60]

*GUCY2C* ON-TARGETplus SMARTpool (Chicago, IL) transfection was performed with pre-confluent HT-29 and Caco-2 cells growing on Trans-well inserts (Becton Dickinson, Bedford, MA) according to manufacturer's instructions. Groups with transfection reagents only (TRAN) were chosen as controls for RNA interference. For subsequent experiments we used monolayers of Caco-2 or HT-29 cells (21 days post-confluent). The medium bathing the apical surface of the monolayers was replaced with 200 µL of DMEM complete medium containing FITC-Dextran (FD-4, Sigma) at 25 mg/mL. The medium bathing the basolateral surface was replaced with 500 µL of DMEM complete medium alone or DMEM supplemented with or without IFNγ (10 ng/ml) and TNFα (10 ng/ml). Fluorescence in basolateral bathing medium was measured using a fluorescence spectrophotometer (Biotek Instruments, VT). The permeability of the monolayer was expressed as a clearance (C; nl·cm*^−2^*·h*^−1^*). Transepithelial electrical resistance (TEER) was measured by E-VOM instrument (World Precision Instruments, Sarasota, FL). Results were expressed as Ohm/cm^2^.

### Measurements of Cytokines

Blood was directly collected from heart through diaphragm and serum was prepared and used to measure cytokines and chemokines with Bioplex ™ [52].

### FACS analysis [61], [62]

Briefly, mouse jejunum was everted and incubated in calcium-magnesium-free HBSS with 1 mM EDTA for 30 minutes at 37°C with gentle shaking to liberate IEC. Cell survival was determined with an Annexin V kit (eBioscience). CD3-/7-AAD- (eBiosciences) was used as a marker for intra-epithelial T lymphocytes. Data were analyzed using FlowJo software.

### Statistical Analysis

Results are presented as the mean ± SEM. Data were analyzed using analysis of variance, 2-tailed Student's *t* test, and the Mann-Whitney test as appropriate (Prism, GraphPad, San Diego, CA). *P* values≤0.05 were considered significant.

## Supporting Information

Figure S1 **Reduction of GCC signaling leads to hyperpermeability in IEC monolayer.** HT-29 IEC monolayers were grown on Transwell filters. Paracellular permeability in post-confluent HT-29 cell monolayers was assessed by the apical-to-basolateral flux of FD4 in the presence and absence of GCC siRNA, n = 5. Results are shown as the mean ± SEM.
(TIF)

Figure S2 **Reduction of GCC signaling leads to hyperpermeability in IEC monolayer.** HT-29 IEC monolayers were grown on Transwell filters. Paracellular permeability in post-confluent HT-29 cell monolayers was assessed by TEER in the presence and absence of GCC siRNA, n = 5. Results are shown as the mean ± SEM.
(TIF)
