# Supplementary figures and images for: Loss of Guanylyl Cyclase C (GCC) Signaling Leads to Dysfunctional Intestinal Barrier
Source: PLoS One. 2011 Jan 31;6(1):e16139. doi: 10.1371/journal.pone.0016139 (PMC3031533; doi:10.1371/journal.pone.0016139)

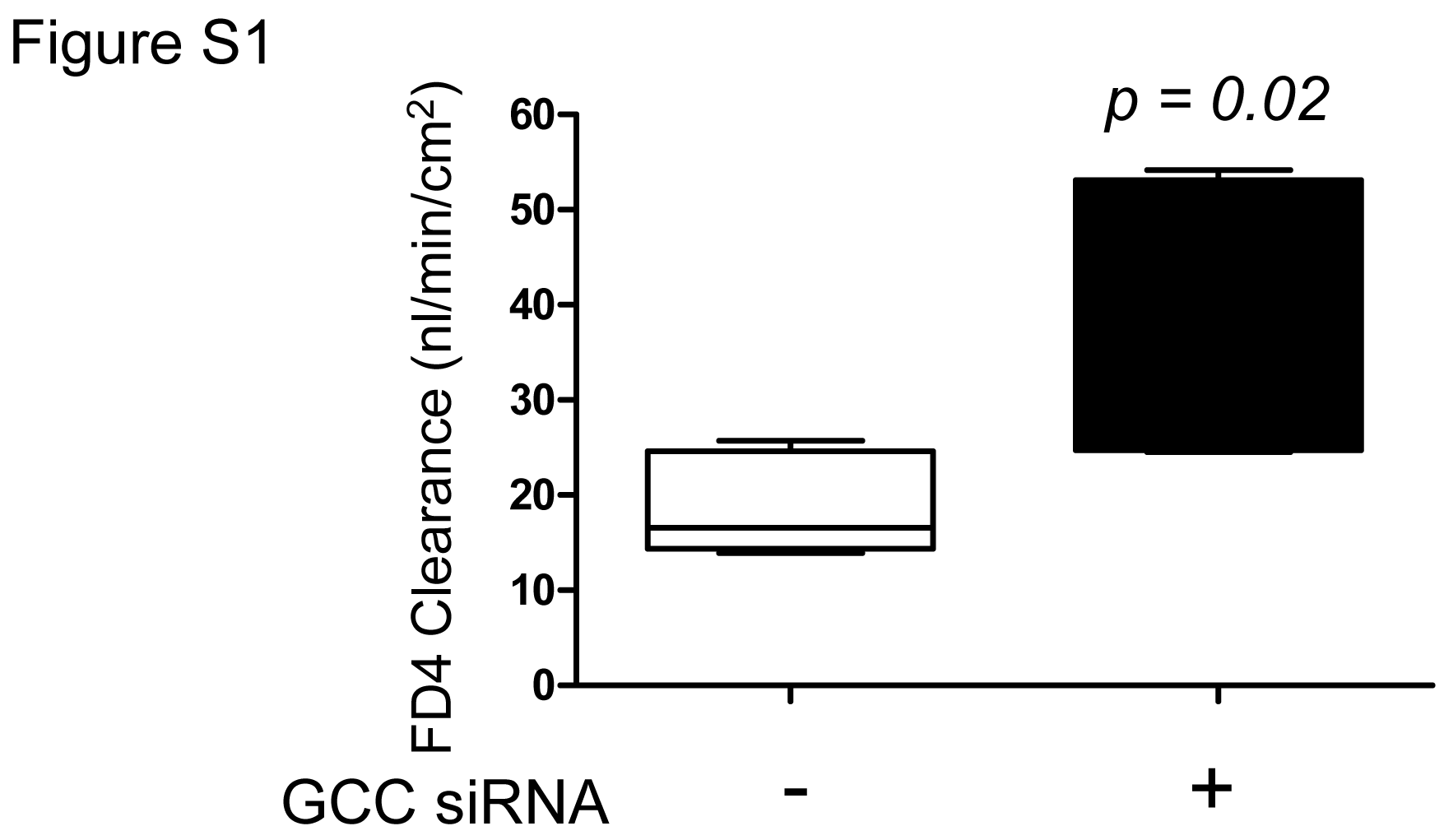

Supplement: Figure S1 — Reduction of GCC signaling leads to hyperpermeability in IEC monolayer. HT-29 IEC monolayers were grown on Transwell filters. Paracellular permeability in post-confluent HT-29 cell monolayers was assessed by the apical-to-basolateral flux of FD4 in the presence and absence of GCC siRNA, n = 5. Results are shown as the mean ± SEM. (TIF) [file pone.0016139.s001.tif]

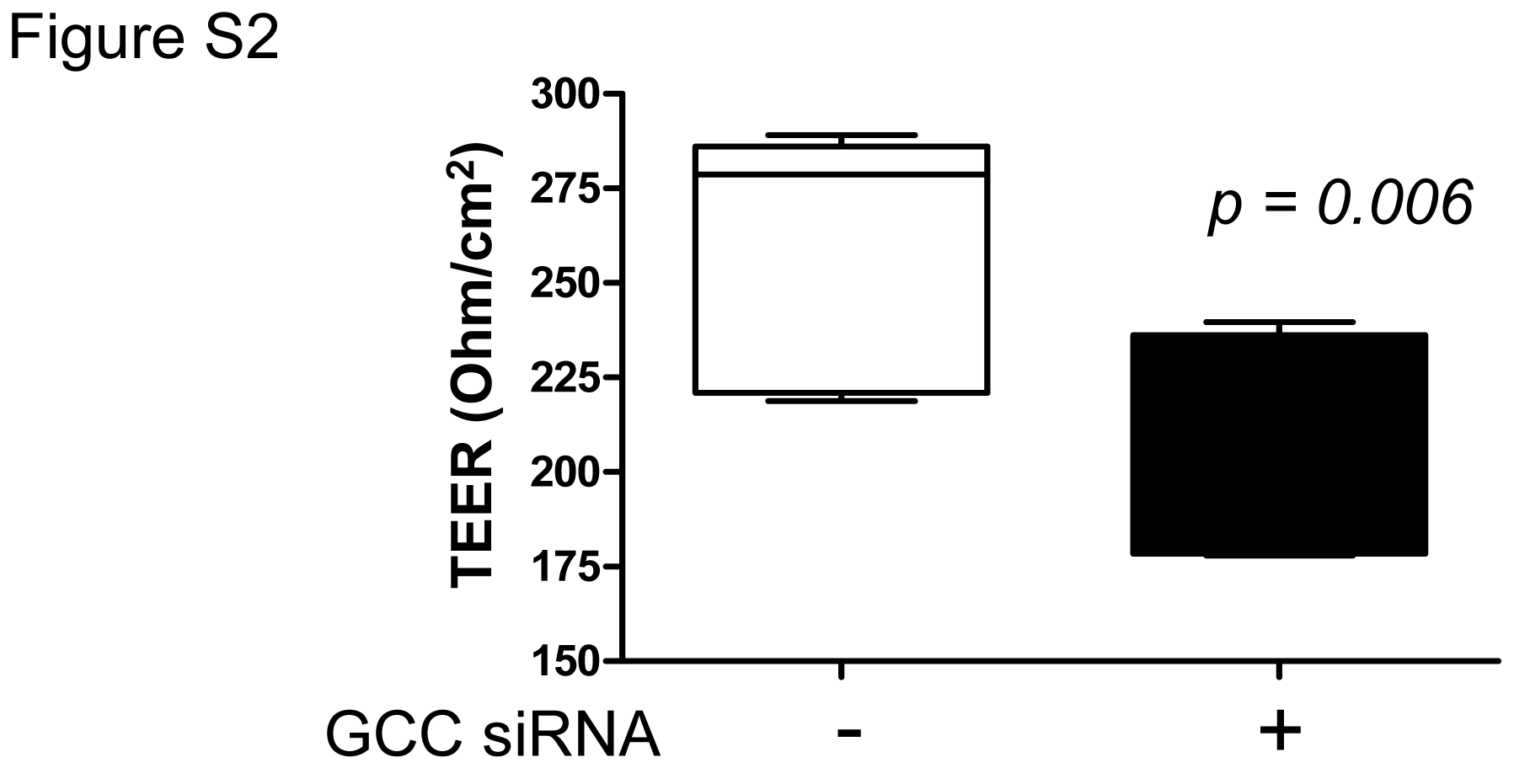

Supplement: Figure S2 — Reduction of GCC signaling leads to hyperpermeability in IEC monolayer. HT-29 IEC monolayers were grown on Transwell filters. Paracellular permeability in post-confluent HT-29 cell monolayers was assessed by TEER in the presence and absence of GCC siRNA, n = 5. Results are shown as the mean ± SEM. (TIF) [file pone.0016139.s002.tif]
